# Lithium Enhances Survival Bias Associated with the Lateral Habenula Suppression

**DOI:** 10.1007/s12264-026-01683-6

**Published:** 2026-07-24

**Authors:** Kunlin Liu, Rui Zhu, Haoyue Guo, Jinghuan Li, Qianfu Wang, Yi He, Chenggang Zhu, Zheng Ma, Xiaodan Jiang, Jack van Honk, Jin Bao

**Affiliations:** 1 Neurosurgery Center, National Key Clinical Specialty, Engineering Research Center of Diagnostic and Therapeutic Technology and Devices for Cerebrovascular Diseases, Ministry of Education, Guangdong Provincial Key Laboratory on Brain Function Repair and Regeneration, Zhujiang Hospital Institute for Brain Science and Intelligence, Zhujiang Hospital, Southern Medical University, or The Second School of Clinical Medicine, Southern Medical University, Guangzhou, 510280 China; 2 Shenzhen Neher Neural Plasticity Laboratory, Shenzhen-Hong Kong Institute of Brain Science, Shenzhen Institutes of Advanced Technology, Chinese Academy of Sciences, Shenzhen, 518055 China; 3 School of Life Sciences, Nanjing University, Nanjing, 210023 China; 4 University of Chinese Academy of Sciences, Beijing, 100049 China; 5 State Key Laboratory of Biomedical Imaging Science and System, Shenzhen Institutes of Advanced Technology, Chinese Academy of Sciences, Shenzhen, 518055 China; 6 Wuhan Weierkang Psychiatric Hospital, Wuhan, 431400 China; 7 Department of Psychology, Utrecht University, 3584 CS Utrecht, The Netherlands; 8 Department of Psychiatry and Mental Health, University of Cape Town, Cape Town, 7925 South Africa; 9 Shenzhen University of Advanced Technology, Shenzhen, 518107 China; 10 College of Biomedical Engineering & Instrument Science, Zhejiang University, Hangzhou, 310027 China

**Dear Editor,**

 In clinical psychiatry, lithium is a primary mood stabilizer with well-established anti-suicidal efficacy [1]. Suicide is a leading cause of mortality among individuals with psychiatric disorders and represents a complex behavioral phenotype involving valuation, decision-making, and impulsivity. Despite decades of clinical use and robust evidence that lithium reduces suicide risk, the mechanisms underlying its anti-suicidal effects remain poorly understood.

Here, we used a translational risky-foraging paradigm in mice to examine how lithium modulates decision-making, when animals must weigh survival probability against immediate reward. To model a clinically relevant mood disorder state, we assessed survival-related decision-making in mice following exposure to a 14-day chronic unpredictable mild stress (CUMS) paradigm. In the risky-foraging task (RFT), the animals face a conflict between a strong drive to feed and a potential predator threat presented by fox urine concentrated in the foraging zone (Fig. 1A). Representative trajectories highlighted the distinct spatial avoidance behaviors exhibited in the lithium-treated group (Fig. 1B). The total distances traveled in the test cage were comparable between the two groups, indicating that locomotion was not affected by lithium (Fig. 1C). However, lithium-treated mice spent significantly less time in the foraging zone (Fig. 1D) and consumed significantly less food compared to control mice (Fig. 1E), indicating that lithium treatment biases action selection toward threat avoidance and increased survival probability.

**Fig. 1:**
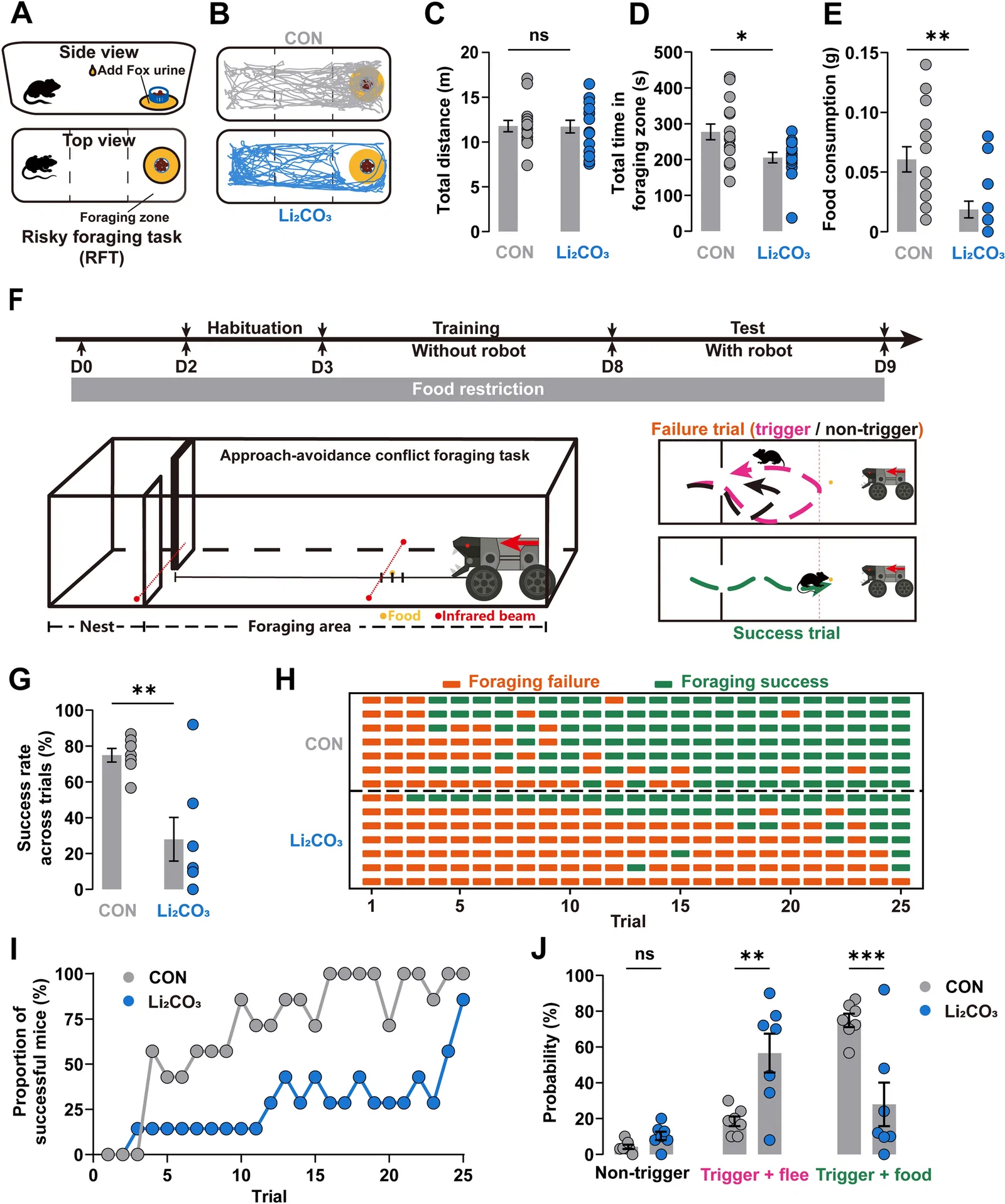
Lithium promotes avoidance behavior and enhances survival probability. **A** Schematic of the risky-foraging task (RFT). **B** Representative trajectories of mice in the RFT. **C** Total distance traveled (*n =* 15 per group, *P* = 0.9542), **D** total time spent in the foraging zone (*P* = 0.0164), and **E** food consumption in the RFT (*P* = 0.0011). **F** Experimental timeline of the approach–avoidance conflict foraging task (top), schematic of the task (bottom left), and three types of behavior in the test session (bottom right). **G** Success rate across trials in the test session (*n =* 7 per group, *P* = 0.0031). **H** Trial-by-trial plot illustrating foraging failure (orange) and success (green) for individual mice. **I** The proportion of mice that successfully retrieved food in each trial. **J** The probability of exhibiting each type of behavior classified as ‘Non-trigger’ (*P* = 0.8849), ‘Trigger + flee’ (*P* = 0.0018), and ‘Trigger + food’ (*P* = 0.0002) was calculated for each mouse. Data are presented as mean ± SEM. *P < 0.05, **P < 0.01, ***P < 0.001 (Unpaired Student’s *t*-test or Mann-Whitney U test, as appropriate for **C–E**, **G**; two-way ANOVA, Šídák multiple comparison *post hoc* tests for **J**). ns, not significant.

Chronic stress is a major contributor to mood disorders and cognitive impairments, including disrupted risk assessment and decision-making [2]. To test whether the lithium-induced action bias is secondary to its amelioration of stress-related affective states, we evaluated behavior in stress-naïve mice. We employed an approach-avoidance conflict foraging task to probe decision-making under repeated reward-threat conflict, in which animals chose between approaching food and avoiding threat on a trial-by-trial basis [3]. In this paradigm, the predator stimulation from a mechanical toy robot is triggered by the approaching action of the foraging animal, and the animals have a nest zone to safely avoid the threat (Fig. 1F). Three types of behaviors were observed in the test session: (1) approaching and successfully triggering the robot, but escaping without food (Trigger + flee); (2) escaping before triggering the robot (Non-trigger); (3) successful food foraging despite triggering the movement of robot (Trigger + food). Consistent with the observation from the risky-foraging task (Fig. 1D and E), the lithium-treated group had a lower success rate in obtaining food reward across multiple trials (Fig. 1G). A trial-by-trial analysis of foraging outcomes revealed distinct behavioral patterns between the two groups: while the control mice rapidly adapted to the threat and transitioned to successful foraging, the lithium-treated group exhibited persistent foraging failures (Fig. 1H), implying a strong and long-lasting action bias toward avoidance (Fig.1I). Across the three behavioral categories, group differences emerged primarily in trials where the robot was triggered. When decisions were made under imminent threat, in these trials, the lithium-treated group predominantly adopted the “flee” strategy (Fig. 1J). These results demonstrate that lithium induces a sustained bias in action selection toward survival prioritization under reward-threat conflict, independent of prior stress exposure.

To rule out the possibility that the effect of lithium on action selection in this reward-threat conflict arises from changes in fear response and/or reward valuation, we first tested the fear response of animals upon visual looming stimulation [4] (Fig. S1A). There is no difference in the latency to escape and the duration staying in the nest between the two groups (Fig. S1B and C). In the Y maze test, animals need to choose between the “High Reward” arm (HR) and the “Low Reward” arm (LR), which is discriminated by the amount of food (Fig. S1E). All animals preferred the high reward following training; thus, lithium treatment did not alter the perception of reward value. When a barrier was placed in front of the food plate in the high-reward arm, the selection rate for the high reward gradually decreased when the height of the barrier increased. The proportion of high reward selection remained the same between the control and the lithium-treated group (Fig. S1F). Based on these results, the lithium-induced increase in risk avoidance cannot be attributed solely to heightened fear responses or to deficits in reward valuation.

The lateral habenula (LHb) is a critical hub for processing aversive signals and regulating survival behaviors [5–8]. Pathological hyperactivation of the LHb due to stress exposure drives behavioral despair and alters defensive behavior to threat [9, 10]. Suppression of LHb activity has been shown to facilitate active escape and risk avoidance, enabling the organism to engage in proactive defensive maneuvers [9, 11, 12]. The LHb receives inputs from brain areas that integrate sensory inputs (thalamus and cortex) and evaluate valence (lateral hypothalamus, amygdala, bed nucleus of the stria terminalis). The LHb makes bidirectional connections with brain areas that adjust motivation (ventral tegmental area and raphe nuclei) and action selection (afferent: basal forebrain; efferent: the periaqueductal gray), which places it at a position to tune motivational drive for behavior [10]. Therefore, the LHb should play an important role in mediating action selection under threat. To determine whether lithium concomitantly altered LHb activity while promoting threat avoidance, we performed *in vivo* multi-channel electrophysiological recordings of LHb neurons in freely moving mice (Fig. 2A). The placements of electrodes were later confirmed *via* magnetic resonance imaging (MRI) (Fig. 2B). At rest, the spontaneous single-neuron spiking activity was significantly reduced in the lithium-treated group compared to that of the control group (Fig. 2C and D), providing a cellular basis for the enhanced avoidance behavior induced by lithium.

**Fig. 2:**
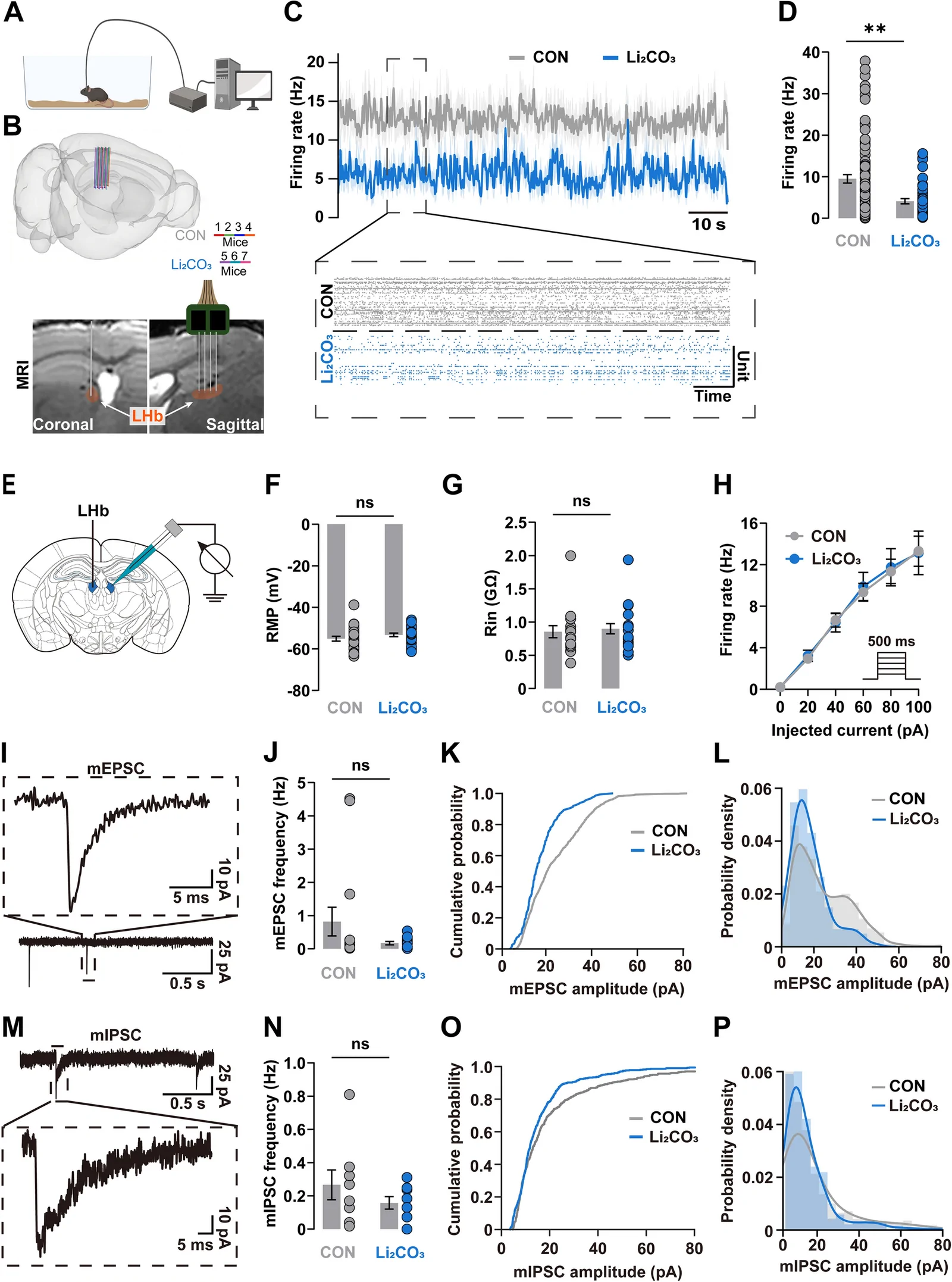
Lithium reduces the firing rate of LHb neurons *in vivo* and decreases the amplitudes of unitary synaptic inputs to LHb neurons. **A** Schematic of *in vivo* multi-channel electrophysiological recordings in freely moving mice. **B** Diagram of the electrode arrays targeting the lateral habenula (top) (4 mice in the control group, 3 mice in the lithium-treated group) and representative magnetic resonance imaging (MRI) confirming the electrode placement (bottom). **C** Mean firing rate of all units from each group (top) (92 units from 4 mice of the control group; 43 units from 3 mice of the lithium-treated group) and representative raster plots of spiking activity (bottom). **D** The mean firing rate of LHb neurons (*P* = 0.0026). **E** Schematic of *in vitro* whole-cell patch-clamp recordings. **F** Resting membrane potential (RMP) (25 cells from 3 mice of the control group; 21 cells from 3 mice of the lithium-treated group, *P* = 0.2308) and **G** input resistance (Rin) (Control: *n =* 16 cells; Lithium-treated: *n =* 19 cells, *P* = 0.5893). **H** Evoked firing frequency in response to depolarizing current steps (from 0 to 100 pA) (Control: *n =* 20 cells; Lithium treated: *n =* 21 cells). **I** A representative trace of miniature excitatory postsynaptic currents (mEPSCs). **J** Mean frequency of mEPSC (Control: *n =* 14 cells; Lithium treated: *n =* 12 cells, *P* > 0.9999), **K** cumulative probability (*P* < 0.0001), and **L** probability density of mEPSC amplitude. **M** Representative traces of miniature inhibitory postsynaptic currents (mIPSCs). **N** Mean frequency of mIPSC (Control: *n =* 8 cells; Lithium treated: *n =* 9 cells, *P* = 0.2641), **O** cumulative probability (*P* < 0.0001), and **P** probability density of mIPSC amplitude. Data are presented as mean ± SEM. **P < 0.01 [Unpaired Student’s t-test or Mann-Whitney U test, as appropriate for **D**, **F–G**, **J**, **N**; two-sample Kolmogorov–Smirnov (K–S) test for **K**, **O**]. ns, not significant.

To investigate the mechanisms underlying the reduced LHb activity following lithium treatment, we performed whole-cell patch-clamp recordings in acute brain slices (Fig. 2E). We first examined the intrinsic membrane properties of LHb neurons. There were no significant differences in either resting membrane potential (RMP) (Fig. 2F) or the input resistance (Rin) (Fig. 2G) between the two groups. Furthermore, the evoked firing frequencies in response to depolarizing current steps remained unchanged (Fig. 2H). These results indicate that lithium did not change the intrinsic excitability of LHb neurons.

Next, we assessed synaptic inputs by recording miniature excitatory postsynaptic currents (mEPSCs) (Fig. 2I) and miniature inhibitory postsynaptic currents (mIPSCs) (Fig. 2M), the quantal size of glutamate and GABA release, respectively. The mean frequencies of mEPSCs and mIPSCs had no significant differences between groups (Fig. 2J and N). However, the cumulative distribution curve of both mEPSC and mIPSC amplitudes exhibited a leftward shift for neurons in the lithium-treated group relative to the distribution from neurons in the control group (Fig. 2K and O). Consistently, the probability density of mEPSC amplitudes showed a shift in the ratio between low-amplitude mEPSCs and high-amplitude ones (Fig. 2L). The Gaussian kernel density estimation suggests that there are distinct groups of synaptic quantal release onto LHb neurons, corresponding to the anatomically distinct excitatory input sources to the LHb [10]. For mIPSCs, there are no obvious multiple Gaussian distributions in the quantal size, and the effect of reduced quantal size was much smaller compared to that of mEPSCs (Fig. 2P). These results indicate that lithium remodels LHb synapses predominantly by reducing the amplitude of excitatory inputs.

In summary, we identified a previously unrecognized role of lithium in modulating action selection under survival threat, extending its functional profile beyond its classical characterization as a mood stabilizer. Notably, the lithium-induced survival-oriented bias was observed across both stress-naïve and chronically stressed animals, indicating a state-independent mechanism. Mechanistically, this effect is associated with a preferential attenuation of excitatory synaptic input onto LHb neurons, resulting in a net reduction of circuit drive. This form of synaptic remodeling is a critical finding, as it moves beyond simple neuromodulation to suggest a more enduring change in the LHb microcircuit. It links the conceptual framework of lithium’s therapeutic action on intracellular signaling pathways, such as GSK-3 and inositol monophosphatase inhibition [13], with a systems-level mechanism involving synaptic plasticity and network reconfiguration

For clinical relevance, these findings position lithium as a modulator of value-based decision-making along the reward-stress axis, which has been proposed as the state switch trigger in the emotional transition of bipolar disorder [14]. The LHb has been studied intensively in animal models as the key hub for stress responses and mediating depressive phenotypes [10]. However, it is difficult to monitor the activity of the LHb in humans due to its small size and deep brain location. Human brain imaging studies have shown that lithium is potent in modulating brain activity in the prefrontal cortex, cingulate cortex, and amygdala in response to emotional stimuli [15]. These brain areas all connect to the LHb *via* direct or indirect pathways. To incorporate our findings that lithium reduces the synaptic input drive and the neuronal firing rate of the LHb, further studies that measure neuronal activity from multiple brain areas are necessary to build an inclusive model for understanding how lithium takes effect in a larger brain network.

## Supplementary Information

Below is the link to the electronic supplementary material.

Supplementary file1 (PDF 198 kb)
