# Supplementary material for: Lithium Enhances Survival Bias Associated with the Lateral Habenula Suppression
Source: Neurosci Bull. 2026 Jul 24;42(10):2399–404. doi: 10.1007/s12264-026-01683-6 (PMC13642231; doi:10.1007/s12264-026-01683-6)
Supplement: Supplementary file 1 [file 12264_2026_1683_MOESM1_ESM.pdf]

Supplementary Materials

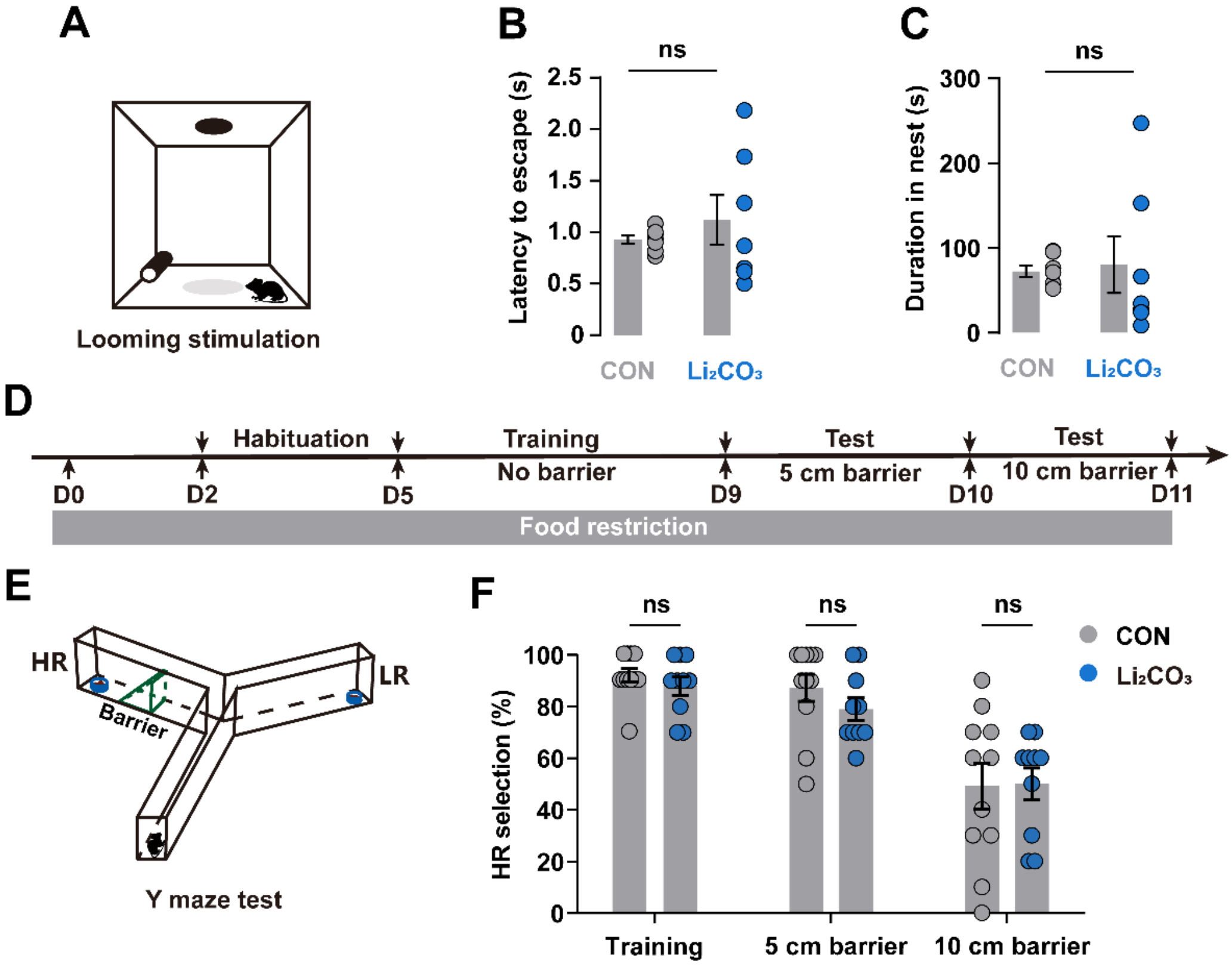

Fig. S1 Lithium does not affect fear responses and reward valuation. **A** Schematic of the looming stimulation. **B** Latency to escape (n = 7 per group, $P = 0.9015$) and **C** duration in nest (n = 7 per group, $P = 0.3829$) of the control and lithium-treated mice in the looming stimulation. **D** Experiment timeline and **E** schematic of the Y maze test. **F** High reward arm selection rate with different barrier heights in control (n = 11) and lithium-treated (n = 10) mice. ($P = 0.5467$ for Training, $P = 0.1759$ for 5 cm barrier, $P = 0.8481$ for 10 cm barrier). Data are presented as mean ± SEM. (Unpaired Student's *t*-test or Mann-Whitney U test, as appropriate). ns, not significant.
